# Self-reported infections in Norway: a study of incidence, doctor visits, antibiotic use, and absence from work

**DOI:** 10.1186/s12875-026-03478-0

**Published:** 2026-07-17

**Authors:** Ingrid K. Rebnord, Knut Erik Emberland, Ingeborg Forthun, Siri Waage, Guri Rortveit, Bjørn Bjorvatn

**Affiliations:** 1 Department of Global Public Health and Primary Care, University of Bergen, Årstadveien 17, Bergen, N-5009 Norway; 2 Department of Disease Burden, Norwegian Institute of Public Health, Bergen, Norway; 3 Norwegian Competence Center for Sleep Disorders, Haukeland University Hospital, Bergen, Norway; 4 Norwegian Institute of Public Health, Oslo, Norway

**Keywords:** Primary healthcare, Respiratory tract infections, Incidence, Antibiotics, Sickness absenteeism, PraksisNett

## Abstract

**Background:**

Infections are common and frequently lead to doctor visits, use of antibiotics, and absence from work.

**Objective:**

To investigate frequency of different self-reported infections and the impact on doctor visits, antibiotic use, and absence from work.

**Method:**

A cross-sectional study based on an online survey in Norwegian primary care, focusing on infections. Individuals between 25 and 70 years of age, were recruited by general practitioners (GPs) through the Norwegian Primary Care Research Network. The respondents reported different types of infections they had experienced in the last 3 months, and if the infection resulted in a GP visit, use of antibiotics or absence from work. Descriptive analyses were used to estimate proportions and multivariate logistic regressions to assess predictors for absence from work.

**Results:**

In total, 74.5% of the respondents reported to have experienced an infection during the last 3 months. Respiratory infections were reported by 63.7%, among these cases, 50.9% reported common cold (95% CI 47.6–54.2), 31.5% influenza-like illness (95% CI 28.4–34.5) and 17.9% COVID-19 (95% CI 15.2–20.5). Among those reporting respiratory tract infections, only 18.6% (95% CI 16.4–20.8) visited a GP. Bronchitis/pneumonia most often led to a GP visit (52.8%, 95% CI 36.3–71.0) and antibiotic prescription (25.0%, 95% CI 11.5–40.7). Respiratory tract infections and gastrointestinal infections had the greatest impact on sickness absence from work. Of these, COVID-19 was the strongest predictor for absence from work (OR 9.29, 95% CI 5.8–14.8).

**Conclusion:**

Respiratory tract infections were the most common type of self-reported infection, and while only one in five visited the GP, the impact on work absence was large. COVID-19 gave the greatest risk of absenteeism.

**Supplementary Information:**

The online version contains supplementary material available at https://doi.org/10.1186/s12875-026-03478-0.

## Introduction

In high-income countries, mortality from infectious diseases has declined dramatically over the past century, largely due to the introduction of vaccines, improved infection control measures, and the use of antibiotics for both treatment and prevention of serious infections [1]. Nevertheless, infections remain a common and important reason for contact with primary care physicians [2, 3]. Typical motivations for seeking medical attention include assessing the severity of symptoms and determining the need for antibiotics or symptom-relieving treatment. Additionally, in Norway—as in several other countries—a physician’s confirmation is required for absence from work or school, particularly when the absence exceeds three days.

In Norway, infections are among the most important causes of short-term absence. During the pandemic, the sickness absence rate was especially high and increasing with a top in 2022, but in contrast to other Nordic countries it had no decrease in 2023 and continued to be higher than before the pandemic [4, 5]. The extent and duration of work absence due to infection vary depending not only on the severity of the illness but also on the nature of the individual’s occupation and the contagiousness of the infection. Individuals working near vulnerable patient populations are generally expected to refrain from attending work while experiencing a contagious illness. In contrast, those with the possibility to work remotely are more likely to continue working despite having mild to moderate symptoms. Respiratory tract infections (RTIs) are the most common reason for consultations in primary care globally [3]. Seasonal epidemics contribute to substantial variation in incidence throughout the year, and the true burden of RTIs is difficult to estimate, as consultation rates do not necessarily reflect actual infection rates [6].

Gastrointestinal infections, which may be foodborne or transmitted through person-to-person contact, represent a significant cause of morbidity and mortality worldwide [7]. In high-income countries, the rate of primary care consultations for these infections is relatively low compared to their actual prevalence in the general population [8, 9].

Urinary tract infections (UTIs) are typically endogenous and occur frequently, particularly among women, older adults, and immunocompromised individuals [10]. UTIs are a common reason for healthcare contact and constitute a major indication for antibiotic prescriptions, as they are usually caused by bacterial pathogens [11, 12].

The aim of the present study was to investigate the incidence of different self-reported infections and how the different infections impact doctor visits, antibiotic use, and sickness absence.

## Materials and methods

### Inclusion and procedures

This cross-sectional study included individuals between 25 and 70 years of age who completed an online survey focusing on infections and sleep. The Norwegian Primary Care Research Network– PraksisNett (www.praksisnett.no) was used to identify eligible participants for recruitment. An earlier publication has described the inclusion in more detail [13]. The present study is based on secondary analyses of parts of the materials focusing on infections and was developed for this study (see Additional file 1).

All Norwegian residents are offered to be enlisted with a general practitioner (GP). GPs participate in Praksisnett on a voluntary basis, those connected are scattered around Norway, and a total of 29 GPs participated in the current study by sending out invitations to eligible individuals. The Praksisnett IT infrastructure, called the Snow system, is able to randomly select patients from the GP patient list based on age and/or diagnoses, but it does not store any information outside of the GP clinic, ensuring anonymity of patients [14]. Each participating GP invited at least 60 individuals, where one of three should have been diagnosed with a sleep problem during the last year (diagnosed with P06 Sleep disturbance) in the International Classification of Primary Care, 2nd edition – ICPC-2), and two of three invited with no P06 diagnose last year. The GPs decided which patients to invite from the lists, and they were instructed to exclude patients who they considered not eligible for participation (e.g., due to terminal disease, acute life crisis etc.).

The GPs used www.helsenorge.no to invite the patients digitally by sending an invitation with the survey link. Helsenorge.no is the official website for information and access to health services for residents of Norway. The invited individuals responded to the invitation by clicking on the link and thereby entering the online survey (provided by Surveyxact by Ramboll). After giving informed consent to participate in the study, they received the survey. The inclusion was done between March 2022 and January 2023.

### Variables

The following socio-demographic information was collected: sex (male; female; prefer not to disclose/other), age (categorized as 25–40; 41–55; 56–70), marital status (single; married/cohabiting; divorced/separated; widow/widower), country of birth (Norway; other European country; Asia; Africa; America; Oceania), children living at home (no; yes), educational level (primary school; secondary school; college/university). Furthermore, the participants reported whether they had experienced the following infections during the last three months (no; yes): common cold, throat infection, ear infection, sinusitis, pneumonia/bronchitis, COVID-19, influenza-like illness, skin infection (erysipelas, herpes labialis, etc.), gastrointestinal infection with vomit and/or diarrhea, urinary tract infection (cystitis, pyelonephritis), venereal disease (chlamydia, genital herpes), and eye infection. Respiratory tract infection (RTI) was defined as reporting common cold, influenza-like illness, COVID-19, throat infection, ear infection, sinusitis or bronchitis/pneumonia, where bronchitis/pneumonia was defined as a lower RTI and the rest defined as upper RTI. The participants also reported whether the specific infection had resulted in a doctor’s visit, prescribed antibiotics, whether the infection had caused absence from work and if so, how many days with absence.

The participants were asked to respond (no; yes) to questions about the following chronic diseases: diabetes, high blood pressure, cardiovascular diseases, neurological diseases, lung diseases, rheumatic diseases, cancer, allergies or sleep apnea.

### Statistical analysis

The data was analysed using descriptive analysis calculating proportions with 95% CI. Regression analyses were conducted using logistic regression estimating odds ratio (OR) with 95% confidence interval (CI) with sickness absence due to an infection as dependent variable, and the different infections as predictors. Both crude and adjusted (for sex, age, marital status, country of birth, children living at home, educational level, and chronic diseases) regressions were conducted. For all statistical analyses, IBM SPSS Statistics 28 was used.

### Ethics

The study was approved by the Regional Committee for Medical and Health Related Research Ethics (REK South-East, application number 268 606). Only participants who provided informed consent received the survey questions.

## Results

A digital invitation was sent to 2493 patients, and 1023 consented to participate, of which 988 completed the questionnaire (39.6% response rate). Thereafter 93 patients were excluded because of missing data for all outcome variables about infections, leaving 895 patients for further statistical analyses. Table 1 presents the background characteristics. The proportion of women was 60%, 90% were Norwegian, and 68% had higher education.

**Table 1:** Distribution of background variables in the study population collected from March 2022 until January 2023 (*n* = 895)

Variables	Number	%
Sex		
Male	355	39.7
Female	540	60.3
Age (years)		
25–40	241	26.9
41–55	365	40.8
56–70	289	32.3
Marital status		
Single	148	16.5
Married/cohabiting	669	74.7
Divorced/separated	68	7.6
Widow/widower	10	1.1
Country of birth		
Norway	812	90.7
Other European country	64	7.2
Countries outside Europe	19	2.1
Children living at home		
No	533	59.6
Yes	362	40.4
Educational level		
Primary school	48	5.4
Secondary school	235	26.3
College/university	612	68.4
Sickness absence from work last 3 months		
Yes	398	44.5
No	497	55.5
Chronic diseases		
Hypertension	141	16.2
Diabetes	43	4.9
Cardiovascular diseases	36	4.1
Neurological diseases	62	7.1
Lung diseases	67	7.7
Rheumatological diseases	127	14.5
Cancer	28	3.2
Allergies	297	34.0
Sleep apnea	88	10.1

### Infections and antibiotic use

**Fig. 1:**
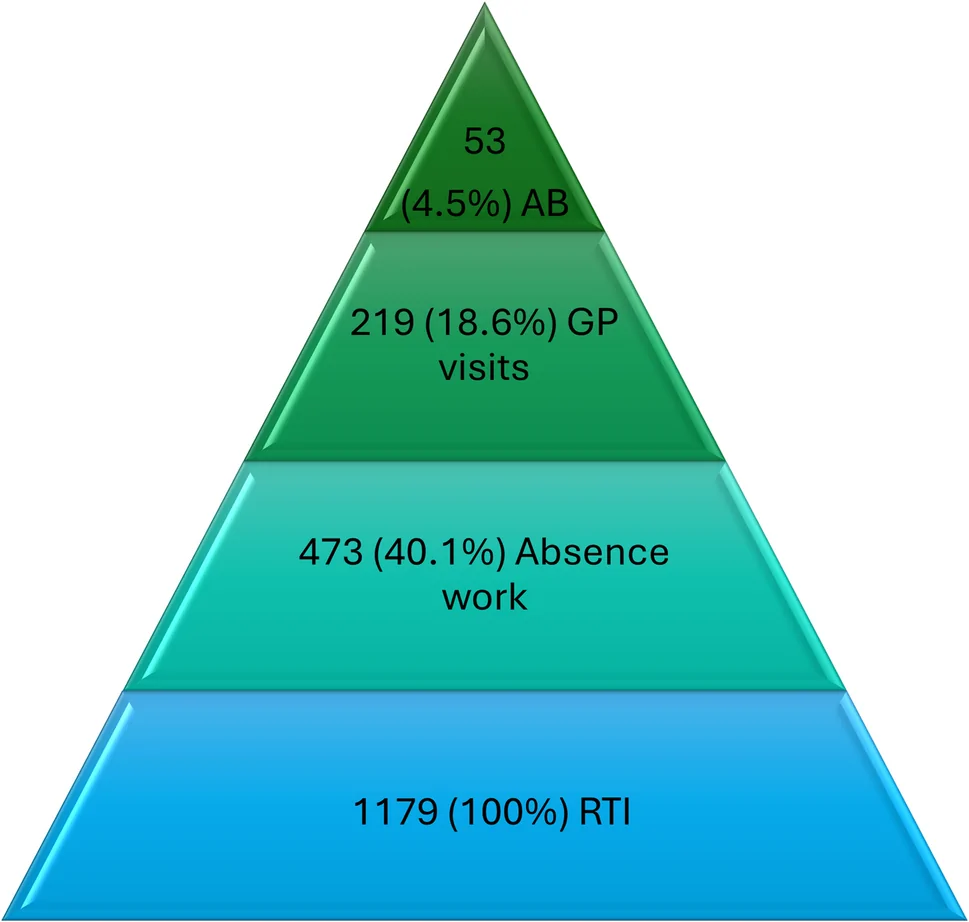
The respiratory tract infection (RTI) clinical “ice-berg”, number of episodes with resulting sickness absence, GP visits and antibiotics (AB) (*n* = 895)

RTIs dominated in number compared to the other infections, 18.6% (95% CI 16.4–20.8) visited their GP, while 4.5% (95% CI 3.3–5.6) used antibiotics for the infection (Fig. 1). Common cold was the most frequent infection, reported by 50.9% of the participants (95% CI 47.6–54.2) (Table 2).

**Table 2:** Distribution of the diagnoses according to frequency, doctor visits, antibiotics and absence from work (*n* = 895)

Diagnoses last 3 months	Total number		Visiting the GP		Antibiotic use		Absence from work	
	*N*	% (95% CI)	*N*	% (95% CI)	*N*	% (95% CI)	*N*	% (95% CI)
Common cold	456	50.9 (47.6–54.2)	59	13.1 (9.7–16.2)	16	3.6 (1.9–5.3)	177	39.4 (34.5–43.3)
Influenza-like illness	282	31.5 (28.4–34.5)	41	15.0 (10.5–18.7)	4	1.5 (0.0–3.0)	98	35.8 (29.2–40.4)
COVID-19	160	17.9 (15.2–20.5)	43	27.2 (20.2–34.4)	5	3.2 (0.6–6.1)	106	67.1 (58.3–73.4)
Throat infection	114	12.8 (10.5–15.0)	23	20.4 (13.1–27.7)	7	6.2 (2.3–10.8)	33	29.2 (20.6–36.9)
Sinusitis	94	10.5 (8.5–12.8)	21	22.6 (14.7–30.9)	3	3.2 (0.0-7.1)	32	34.4 (25.0–44.0)
Ear infection	37	4.1 (3.0-5.6)	13	35.1 (19.4–50.0)	9	24.3 (10.3–37.9)	8	21.6 (8.3–34.1)
Bronchitis/pneumonia	36	4.0 (2.8–5.4)	19	52.8 (36.3–71.0)	9	25.0 (11.5–40.7)	19	52.8 (37.8–69.2)
Skin infection	193	21.9 (19.1–24.6)	41	21.2 (15.4–27.0)	17	8.8 (5.2–13.0)	8	4.1 (1.5–7.1)
Gastrointestinal infection	171	19.1 (16.9–22.3)	26	15.2 (9.3–20.0)	7	4.1 (1.1–6.9)	51	29.8 (21.5–35.5)
Urinary tract infection	48	5.4 (3.9–6.9)	30	62.5 (46.5–74.4)	33	68.8 (52.5–80.0)	6	12.5 (3.5–22.2)
Venereal disease	16	1.8 (1.1–2.8)	8	50.0 (25.0-71.4)	6	37.5 (14.3–60.0)	1	6.3 (0.0–20.0)
Eye infection	71	7.9 (6.4–10.0)	33	46.5 (34.8–56.9)	19	26.8 (16.6–36.8)	12	16.9 (8.6–24.6)
Total	1678		357		135		551	

Gastrointestinal infection and skin infection both occurred in 19.1% (95% CI 16.9–22.3) and 21.9 (95% CI 19.1–24.6) of the respondents while UTI was reported by 5.4 (95% CI 3.9–6.9). Bronchitis/pneumonia, eye infection, venereal disease, and UTI had the highest proportion of doctor visits (Table 2). Antibiotic use was most common for UTI and venereal disease. The mean number of any type of infection during the last three months was 1.9 (95% CI 1.8-2.0) per respondent, but 25.5% reported no infections at all, and 36.3% reported no RTIs. Among those with infections, the mean number of infections was 2.5 (95% CI 2.4–2.7). The 3-month incidence of RTI was 1.3 per person. A logistic regression was performed to identify factors associated with experiencing a RTI (Additional file 2). Having chronic lung disease was significantly associated with RTI, OR 1.98 (95% CI 1.09–3.59), whereas being in age groups above 40 years was associated with reduced odds of RTI, OR 0.52 (95% CI 0.35–0.77). 

### Absence from work

**Fig. 2:**
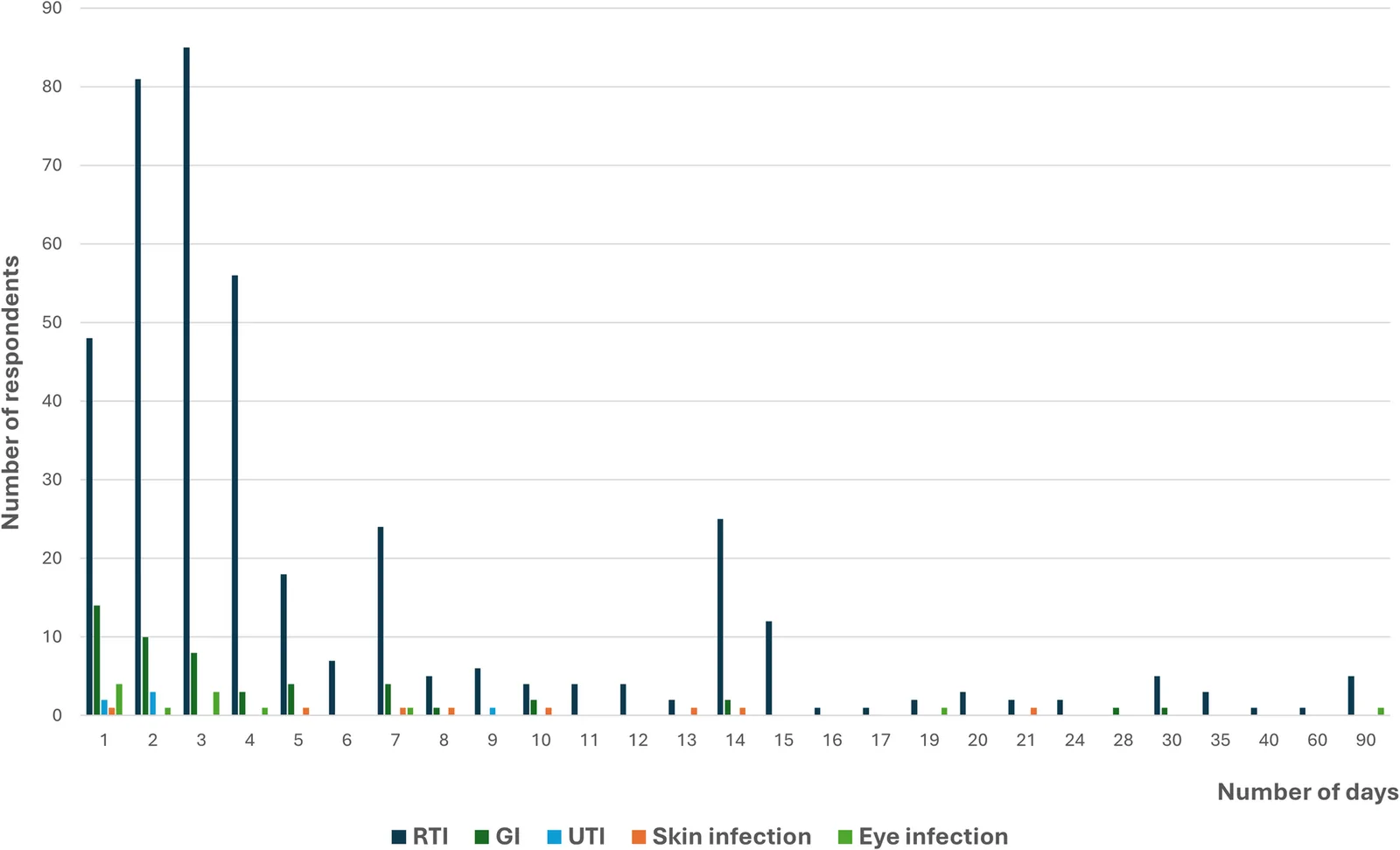
Distribution of number of days with sickness absence from work per diagnoses group* (*n* = 895)

RTIs dominated in terms of absence from work (Table 2) and duration of absence (Fig. 2). Most respondents with RTIs were absent for 7 days (78.4% of those with absence from work). After 14 days, 90.7% were back at work.

Of those reporting an RTI, 40.1% (95% CI 37.3–42.9) had absence from work (Fig. 1).

Different types of respiratory tract infections (RTIs) were associated with varying rates of work absence. Approximately one-third of individuals with upper RTIs or influenza-like illness reported being absent from work. In contrast, 53% of those diagnosed with bronchitis or pneumonia reported work absence. Among participants with COVID-19, two out of three reported sick leave.

However, the duration of absence did not significantly differ across RTI types. The mean duration of absence among those who reported being absent ranged from seven to eight days, apart from throat infections, which had a higher mean duration of 10 days. We found no significant predictors of sickness absence exceeding seven days among individuals with respiratory tract infections, when absence was analyzed as a dichotomous outcome (≤ 7 days vs. >7 days). Notably, among participants who were absent from work for one to three months, 72.0% were women.

Table 3 presents the predictors of sickness absence. In the adjusted analyses, common cold, influenza-like illness, COVID-19, sinusitis, and gastrointestinal infections were significantly associated with increased odds of absence. In contrast, urinary tract infections were associated with lower odds of absence. The strongest predictor for absence was COVID-19 with OR 9.3 (95% CI 5.8–14.8). A similar regression analysis was conducted excluding respondents with children at home. The differences were minor; however, in this subgroup, gastrointestinal infections were no longer significantly associated with work absence (OR 1.60, 95% CI: 0.87–2.94). For respiratory tract infections, the pattern of sickness absence remained consistent, as did the associations with background variables.

**Table 3:** Logistic regression analyses of absence from work because of infections (no = 0, yes = 1) as the dependent variable (*n* = 873)

Variables		Crude OR	Adjusted OR*	*P*-value
		(95% CI Lower-Upper)	(95% CI Lower-Upper)	
Sex	Male	1.00 (Ref.)		
	Female	1.35 (1.02–1.80)	1.13 (0.77–1.65)	0.535
Age	25–40	1.00 (Ref.)		
	41–55	0.62 (0.45–0.85)	0.78 (0.49–1.24)	0.066
	56–70	**0.39 (0.27–0.55)**	**0.35 (0.20–0.62)**	**< 0.001**
Marital status	Single	1.00 (Ref.)		
	Married/cohabiting	0.84 (0.58–1.20)	0.86 (0.52–1.41)	0.551
	Divorced/separated	0.66 (0.36–1.22)	0.74 (0.33–1.66)	0.468
	Widow/widower	0.17 (0.02–1.34)	0.21 (0.02–2.01)	0.185
Country of birth	Norway	1.00 (Ref.)		
	Other European countries	0.94 (0.56–1.57)	0.89 (0.45–1.77)	0.744
	Countries outside Europe	0.16 (0.06–0.43)	0.30 (0.08–1.14)	0.076
Children living at home	No	1.00 (Ref.)		
	Yes	1.13 (1.01–1.74)	1.21 (0.83–1.76)	0.316
Educational level	Primary school	1.00		
	Secondary school	2.21 (0.99–4.92)	2.14 (0.70–6.48)	0.180
	College/university	2.69 (1.24–5.81)	2.92 (0.99–8.56)	0.052
Chronic diseases				
Diabetes	No	1.00 (Ref.)		
	Yes	0.94 (0.49–1.80)	0.91 (0.37–2.25)	0.934
Hypertension	No	1.00 (Ref.)		
	Yes	0.83 (0.56–1.23)	1.12 (0.66–1.90)	0.998
Cardiovascular diseases	No	1.00 (Ref.)		
	Yes	0.74 (0.35–1.56)	1.02 (0.39–2.69)	0.580
Neurological diseases	No	1.00 (Ref.)		
	Yes	0.78 (0.45–1.38)	0.65 (0.31–1.33)	0.653
Lung diseases	No	1.00 (Ref.)		
	Yes	1.26 (0.75–2.10)	1.08 (0.55–2.10)	0.739
Rheumatological diseases	No	1.00 (Ref.)		
	Yes	0.88 (0.59–1.32)	0.90 (0.54–1.50)	0.202
Cancer	No	1.00 (Ref.)		
	Yes	0.77 (0.34–1.78)	1.20 (0.37–3.91)	0.840
Allergies	No	1.00 (Ref.)		
	Yes	1.10 (0.82–1.48)	0.86 (0.59–1.27)	0.805
Sleep apnea	No	1.00 (Ref.)		
	Yes	1.13 (0.71–1.78)	1.07 (0.57–2.01)	0.472
Infections				
Common cold	No	1.00 (Ref.)		
	Yes	**4.79 (3.52–6.50)**	**2.72 (1.81–4.10)**	**< 0.001**
Influenza-like illness	No	1.00 (Ref.)		
	Yes	**4.06 (3.01–5.48)**	**2.52 (1.66–3.82)**	**< 0.001**
COVID-19	No	1.00 (Ref.)		
	Yes	**6.68 (4.58–9.75)**	**9.29 (5.82–14.84)**	**< 0.001**
Throat infection	No	1.00 (Ref.)		
	Yes	2.71 (1.82–4.04)	0.91 (0.52–1.59)	0.733
Sinusitis	No	1.00 (Ref.)		
	Yes	**3.75 (2.41–5.85)**	**2.47 (1.42–4.32)**	**< 0.001**
Ear infection	No	1.00 (Ref.)		
	Yes	1.44 (0.75–2.79)	0.73 (0.31–1.69)	0.457
Bronchitis/pneumonia	No	1.00 (Ref.)		
	Yes	4.06 (2.00-8.24)	2.22 (0.89–5.48)	0.085
Skin infection	No	1.00 (Ref.)		
	Yes	1.58 (1.15–2.14)	1.35 (0.89–2.04)	0.161
Gastrointestinal infection	No	1.00 (Ref.)		
	Yes	**3.16 (2.25–4.43)**	**2.30 (1.48–3.56)**	**< 0.001**
Urinary tract infection	No	1.00 (Ref.)		
	Yes	1.02 (0.56–1.87)	**0.41 (0.18–0.92)**	**0.030**
Venereal disease	No	1.00 (Ref.)		
	Yes	1.75 (0.67–4.59)	1.44 (0.41–4.50)	0.567
Eye infection	No	1.00 (Ref.)		
	Yes	1.58 (0.98–2.57)	1.14 (0.60–2.17)	0.684

*Adjusted for sex, age, marital status, country of birth, children living at home, educational level, chronic diseases and infections

## Discussion

This study revealed substantial variation across infection types in terms of frequency, healthcare utilization, antibiotic use, and work absence. The most frequent infections were the common cold and influenza-like illness. Fewer than one in five individuals experiencing common cold, influenza-like illness, throat infection, sinusitis, skin infection, or gastrointestinal infection consulted a physician.

Antibiotic use was generally low for respiratory tract infections, except for ear infections and bronchitis/pneumonia, for which approximately one in four individuals received antibiotics. The highest rate of antibiotic use was observed for urinary tract infections (UTIs), with 68.8% reporting antibiotic treatment.

Respiratory tract infections and gastrointestinal infections had the greatest impact on work absence. More than one in three individuals with these infections reported being absent from work, with the highest rate observed for COVID-19, where 67.1% of affected individuals reported work absence. Self-reported infections are not widely researched. A comparable study from Great Britain in 2013 introduced the term “the RTI clinical iceberg” as a term illustrating how the high frequency of infections, symptom burden in the population and absence from work is significantly higher than the number of consultations and prescriptions of antibiotics indicate [6]. In Norway, consumption of antibiotics is generally low and has decreased significantly since 2012 [15].The prescription rate in our study from 2022 to 2023 is lower (4.5%, 95% CI 3.3–5.6 ) than in Great Britain in 2013 (7.1%, 95% CI not available). The number visiting GP was the same, 18.6 in Norway (95% CI 16.4–20.8) and 19.7% (95% CI 16.8–22.9) in Great Britain. In Norway you must contact a doctor if you need a sick leave after 3 days of absence from work (for some employees after 8 days). Thus, it is likely that a great number of the GP’s visits were for sick leave certification rather than for antibiotics. The term iceberg is nevertheless very illustrative of the importance and symptom burden of infections, where absence from work accounts for less than half of the infections and prevalence based on patients in need of a doctor and antibiotics is only the tip of the iceberg.

In our material, respiratory infections were by far the most frequent cause of sickness absence, which agrees with what is known from Norwegian sickness absence statistics [16]. There was also a large variation in the duration of the absence, with 90% recovering within 7 days while a small proportion had a prolonged course. Since we have only collected data from last 3 months it is not possible to state if this is due to long-term symptoms after RTI, like long-COVID [17–19]. There was no difference between the different types of respiratory infections and length of absence, but our study confirms what is known from other studies, that a small proportion has a prolonged course after different respiratory tract infections [17]. We found that participants with COVID-19 were more often absent from work compared to other types of infections, and COVID-19 was clearly the strongest predictor of sickness absence also when adjusting for the background variables. Our data cannot determine whether this reflects a greater fear of spreading the infection when knowing that one has this specific virus, or if it indicates that these patients experience a more severe illness compared to other influenza-like illnesses. The same finding is documented in a Greek study from the same period reporting how COVID-19 resulted in more absences among healthcare personnel than influenza [20]. Future studies are needed to clarify whether this has changed after the pandemic and with spreading of other variants of the virus.

We chose to combine bronchitis and pneumonia in the questionnaire, because it is challenging for both clinicians and patients to differentiate between them [21]. In healthy immunocompetent individuals, bronchitis is considered as a viral infection, while pneumonia more often has a bacterial etiology. We expect a predominance of bronchitis in the age group included in the present study [22]. In our material, there were fewer patients reporting bronchitis/pneumonia compared to upper respiratory tract infections, and among those reporting bronchitis/pneumonia, only half had consulted a doctor and one in four had received antibiotics. This means that the prescription rate was around 50% (9 of 19 that visited a doctor). Compared to a previous Norwegian registry study, which found a prescription rate of 36% for acute bronchitis and 65% for pneumonia in 2019, our results were in the middle and reflect the mix of these diagnoses [15]. For the other RTI diagnoses, the proportion visiting GPs and the use of antibiotics were lower. Among participants reporting sinusitis only 23% had visited their GP, and of these, 14% received an antibiotic prescription, which is much lower than in the register-based study [15]. This probably reflects that many patients experience sinusitis symptoms but never visit GP and to a larger extent accept to wait for spontaneous symptom relief.

Gastrointestinal infection was commonly reported in our study with a three-month prevalence of 19%, a figure much higher compared to an incidence of 6%, 8% and 12% of the Norwegian population per year as reported in three population-based studies from Norway [23–25]. Although the proportion of participants with gastrointestinal infection who were treated with antibiotics was relatively low (4.1%), the proportion of participants with gastrointestinal infection treated with antibiotics of those who visited their GP was 26.9%. This is higher compared to two registry-based studies of antibiotic treatment for gastroenteritis consultations in primary care from Norway (1.8%) and The Netherlands (8.8%) [9, 26]. However, this finding is uncertain due to a low number of gastrointestinal infection cases with antibiotic treatment in our study. Furthermore, we cannot rule out that some participants reported gastrointestinal complaints other than gastroenteritis as gastrointestinal infection. Gastrointestinal infections were associated with a high risk of work absence; however, this association was not statistically significant among individuals without children at home, suggesting that children may serve as vectors for such infections.

UTI resulted in more frequent visits to the doctor as antibiotics are expected by patients to a higher extent than for other infections. The proportion of UTI who received antibiotics was 69% in our study. This corresponds well with a previous Norwegian study that found that the proportion who received antibiotics for UTIs were 65% [12]. UTI accounted for a very small proportion of sickness absence, probably due to the high frequency of treatment with antibiotics and the rapid effect of treatment. In the adjusted analyses it seems to protect for absence from work. Patients with UTIs typically present without systemic symptoms at the time of seeking medical care, as consultations often occur early in the course of illness. This may explain why UTIs were associated with lower odds of sickness absence compared to other types of infections.

An association between having young children under the age of seven and experiencing respiratory tract infections (RTIs) or work absence was observed only in the univariate analyses. Parents of children under the age of 13 are entitled to use care days when their child is ill, which may explain why having young children did not increase the risk of work absence.

Older age was a strong predictor of reduced risk of work absence. We do not know for sure why we see this pattern, and there may be many explanations. Notably, our finding aligns with national sick leave statistics from Norway, which show that individuals aged 20–40 have the highest rates of sick leave due to respiratory tract infections [27]. In the unadjusted analyses, respondents with origins outside Europe appeared to have a lower risk of work absence. This is likely attributable to the fact that a smaller proportion of individuals from outside Europe are employed. In unadjusted analyses, also respondents with higher education appeared to have an increased risk of work absence. Although research on short-term sickness absence is limited, a 2015 study examined associations with gender, age, childcare responsibilities, and education level, finding that these associations were generally much weaker for short-term absence, making causal interpretations challenging [28].

### Strengths and limitations

The present study had a response rate of 39.6% which is regarded as acceptable for a study with digital invitations and questionnaire [29]. The patients received the invitation in their public health portal as a message from their GP, and this may have increased the response rate. The portal requires the patient to use a secure login to read the message, and we do not know if some of the invited patients were unable to open this type of message. Based on experience with this age group (25 to 70 years), very few individuals do not use the public health portal, which is also used for online dialogue with their GP [30]. The GPs could actively refrain from inviting patients who they knew were in very poor physical or mental health, but we do not know to what extent this happened. In this age group one would assume that it applies to very few, and we therefore believe that the sample is probably representative of the age group from 25 to 70 years. The study included this age group since we focused on a working population.

Frequent visits to the general practitioner, as well as access to digital consultations, may also contribute to increased rates of sick leave and antibiotic prescriptions. A limitation of our dataset is that we do not have information on the total number of consultations and visits.

Another limitation was that one in three participants were selected because of a sleep diagnosis noted in their medical record. Sleep disorders are a very common in general practice [31, 32], and infections are shown to be more frequent among patients with sleep problems, so we cannot rule out that this has also produced a certain bias in the material, with an over-representation in the frequency of infections [33–35]. In our study population, it is not possible to identify which individuals were included due to being registered with sleep disturbances in their medical records. Therefore, a comparison between the two groups cannot be conducted. However, we have controlled for sleep-related variables in the analysis of absenteeism, and this did not yield any statistically significant results.

All data in the study were based on self-report and this is both a strength and a limitation [36]. The questionnaire dealing with infections has not been validated in previous studies, this constitutes a limitation. Self-reported diagnoses are a weakness, however, one must assume that COVID-19 may be relatively safe due to the possibility of self-testing, while the other infections are probably linked to the patient’s perception of which organ (ears, sinuses or throat etc.) is most affected, and partly to how sick they felt. The study nevertheless gives a good indication of the prevalence of infections in a general population.

Recall bias is another important bias in such a survey. We chose three months because a longer period would entail a greater risk of error in recall [37].

## Conclusion

Four of five RTIs were never reported to a doctor but were managed by self-care, and two of five were absent from work due to such an infection. RTIs had a major impact on sickness absences of up to 2 weeks, while gastrointestinal, skin and UTIs had a negligible impact on sickness absences. COVID-19 posed the greatest predictor of sickness absence.

## Supplementary Information

Additional File 1.

Additional File 2.

## Data Availability

The data underlying this article will be shared at reasonable request to the corresponding author.

## Abbreviations

RTI: Respiratory tract infection
UTI: Urinary tract infection
GP: General practitioner
