# Supplementary material for: Self-reported infections in Norway: a study of incidence, doctor visits, antibiotic use, and absence from work
Source: BMC Prim Care. 2026 Jul 17;27:364. doi: 10.1186/s12875-026-03478-0 (PMC13660301; doi:10.1186/s12875-026-03478-0)
Supplement: Supplementary file 1 [file 12875_2026_3478_MOESM1_ESM.docx]

# SURVEY – INFECTION PART

**1. Gender:** 1. Male 2. Female 3. Prefer not to say/Other

**2. Age**: Dropdown menu starting from 25 years and above

**3. Marital status:** 1. Single 2. Married/Cohabiting 3. Divorced or Separated 4. Widowed

**4. Country of birth:** 1. Norway 2. Another European country 3. Asia 4. Africa 5. America 6. Oceania

**5. Highest level of education completed:** 1. Primary school 2. Secondary school 3. University/College

**6. Do you have children living at home?** 1. No 2. Yes, 0-2 years 3. Yes, 3-6 years 4. Yes, 7-12 years 5. Yes, 13-18 years

**7. Have you had sick leave in the last 3 months due to your own illness?** (Include both sick leave and self-certified sick leave, but do NOT include absence due to illness of children or dependents.)

Yes

No

If yes, estimate the total sick leave in the last 3 months

a. 1-7 days
b. 8-14 days
c. 15-30 days
d. 31-90 days

**8. Have you had the following infections in the last 3 months?**

**8a. Common cold?**

No

Yes

If yes, the following additional questions are asked:

How many times in the last 3 months?

1 time

2 times

3 times

More than 3 times

Have you contacted a doctor for such an infection in the last 3 months?

Yes

No

Unsure

Have you received antibiotics for such an infection in the last 3 months?

Yes

No

Unsure

How many days have you felt sick from such an infection in the last 3 months?

Dropdown from 0 to 90 days

How sick did you feel at the worst?

1 = not sick

2 = slightly sick

3 = somewhat sick

4 = moderately sick

5 = very sick

6 = life-threateningly sick

Did such an infection lead to absence from work/school?

Yes

No

Unsure

If yes, the following additional question is asked:

How many days were you absent?

Dropdown from 1 to 90 days

**8b. Throat infection?**

No

Yes

If yes, the following additional questions are asked:

How many times in the last 3 months?

1 time

2 times

3 times

More than 3 times

Have you contacted a doctor for such an infection in the last 3 months?

Yes

No

Unsure

Have you received antibiotics for such an infection in the last 3 months?

Yes

No

Unsure

How many days have you felt sick from such an infection in the last 3 months?

Dropdown from 0 to 90 days

How sick did you feel at the worst?

1 = not sick

2 = slightly sick

3 = somewhat sick

4 = moderately sick

5 = very sick

6 = life-threateningly sick

Did such an infection lead to absence from work/school?

Yes

No

Unsure

If yes, the following additional question is asked:

How many days were you absent?

Dropdown from 1 to 90 days

**8c. Ear infection?**

No

Yes

If yes, the following additional questions are asked:

How many times in the last 3 months?

1 time

2 times

3 times

More than 3 times

Have you contacted a doctor for such an infection in the last 3 months?

Yes

No

Unsure

Have you received antibiotics for such an infection in the last 3 months?

Yes

No

Unsure

How many days have you felt sick from such an infection in the last 3 months?

Dropdown from 0 to 90 days

How sick did you feel at the worst?

1 = not sick

2 = slightly sick

3 = somewhat sick

4 = moderately sick

5 = very sick

6 = life-threateningly sick

Did such an infection lead to absence from work/school?

Yes

No

Unsure

If yes, the following additional question is asked:

How many days were you absent?

Dropdown from 1 to 90 days

**8d. Sinusitis?**

No

Yes

If yes, the following additional questions are asked:

How many times in the last 3 months?

1 time

2 times

3 times

More than 3 times

Have you contacted a doctor for such an infection in the last 3 months?

Yes

No

Unsure

Have you received antibiotics for such an infection in the last 3 months?

Yes

No

Unsure

How many days have you felt sick from such an infection in the last 3 months?

Dropdown from 0 to 90 days

How sick did you feel at the worst?

1 = not sick

2 = slightly sick

3 = somewhat sick

4 = moderately sick

5 = very sick

6 = life-threateningly sick

Did such an infection lead to absence from work/school?

Yes

No

Unsure

If yes, the following additional question is asked:

How many days were you absent?

Dropdown from 1 to 90 days

**8e. Bronchitis/Pneumonia?**

No

Yes

If yes, the following additional questions are asked:

How many times in the last 3 months?

1 time

2 times

3 times

More than 3 times

Have you contacted a doctor for such an infection in the last 3 months?

Yes

No

Unsure

Have you received antibiotics for such an infection in the last 3 months?

Yes

No

Unsure

How many days have you felt sick from such an infection in the last 3 months?

Dropdown from 0 to 90 days

How sick did you feel at the worst?

1 = not sick

2 = slightly sick

3 = somewhat sick

4 = moderately sick

5 = very sick

6 = life-threateningly sick

Did such an infection lead to absence from work/school?

Yes

No

Unsure

If yes, the following additional question is asked:

How many days were you absent?

Dropdown from 1 to 90 days

**8f. COVID-19?**

No

Yes

If yes, the following additional questions are asked:

How many times in the last 3 months?

1 time

2 times

3 times

More than 3 times

Have you contacted a doctor for such an infection in the last 3 months?

Yes

No

Unsure

Have you received antibiotics for such an infection in the last 3 months?

Yes

No

Unsure

How many days have you felt sick from such an infection in the last 3 months?

Dropdown from 0 to 90 days

How sick did you feel at the worst?

1 = not sick

2 = slightly sick

3 = somewhat sick

4 = moderately sick

5 = very sick

6 = life-threateningly sick

Did such an infection lead to absence from work/school?

Yes

No

Unsure

If yes, the following additional question is asked:

How many days were you absent?

Dropdown from 1 to 90 days

**8g. Influenza-like illness?**

No

Yes

If yes, the following additional questions are asked:

How many times in the last 3 months?

1 time

2 times

3 times

More than 3 times

Have you contacted a doctor for such an infection in the last 3 months?

Yes

No

Unsure

Have you received antibiotics for such an infection in the last 3 months?

Yes

No

Unsure

How many days have you felt sick from such an infection in the last 3 months?

Dropdown from 0 to 90 days

How sick did you feel at the worst?

1 = not sick

2 = slightly sick

3 = somewhat sick

4 = moderately sick

5 = very sick

6 = life-threateningly sick

Did such an infection lead to absence from work/school?

Yes

No

Unsure

If yes, the following additional question is asked:

How many days were you absent?

Dropdown from 1 to 90 days

**8h. Skin infection?**

No

Yes

If yes, the following additional questions are asked:

How many times in the last 3 months?

1 time

2 times

3 times

More than 3 times

Have you contacted a doctor for such an infection in the last 3 months?

Yes

No

Unsure

Have you received antibiotics for such an infection in the last 3 months?

Yes

No

Unsure

How many days have you felt sick from such an infection in the last 3 months?

Dropdown from 0 to 90 days

How sick did you feel at the worst?

1 = not sick

2 = slightly sick

3 = somewhat sick

4 = moderately sick

5 = very sick

6 = life-threateningly sick

Did such an infection lead to absence from work/school?

Yes

No

Unsure

If yes, the following additional question is asked:

How many days were you absent?

Dropdown from 1 to 90 days

**8i. Gastrointestinal infection?**

No

Yes

If yes, the following additional questions are asked:

How many times in the last 3 months?

1 time

2 times

3 times

More than 3 times

Have you contacted a doctor for such an infection in the last 3 months?

Yes

No

Unsure

Have you received antibiotics for such an infection in the last 3 months?

Yes

No

Unsure

How many days have you felt sick from such an infection in the last 3 months?

Dropdown from 0 to 90 days

How sick did you feel at the worst?

1 = not sick

2 = slightly sick

3 = somewhat sick

4 = moderately sick

5 = very sick

6 = life-threateningly sick

Did such an infection lead to absence from work/school?

Yes

No

Unsure

If yes, the following additional question is asked:

How many days were you absent?

Dropdown from 1 to 90 days

**8j. Urinary tract infection?**

No

Yes

If yes, the following additional questions are asked:

How many times in the last 3 months?

1 time

2 times

3 times

More than 3 times

Have you contacted a doctor for such an infection in the last 3 months?

Yes

No

Unsure

Have you received antibiotics for such an infection in the last 3 months?

Yes

No

Unsure

How many days have you felt sick from such an infection in the last 3 months?

Dropdown from 0 to 90 days

How sick did you feel at the worst?

1 = not sick

2 = slightly sick

3 = somewhat sick

4 = moderately sick

5 = very sick

6 = life-threateningly sick

Did such an infection lead to absence from work/school?

Yes

No

Unsure

If yes, the following additional question is asked:

How many days were you absent?

Dropdown from 1 to 90 days

**8k. Venereal disease?**

No

Yes

If yes, the following additional questions are asked:

How many times in the last 3 months?

1 time

2 times

3 times

More than 3 times

Have you contacted a doctor for such an infection in the last 3 months?

Yes

No

Unsure

Have you received antibiotics for such an infection in the last 3 months?

Yes

No

Unsure

How many days have you felt sick from such an infection in the last 3 months?

Dropdown from 0 to 90 days

How sick did you feel at the worst?

1 = not sick

2 = slightly sick

3 = somewhat sick

4 = moderately sick

5 = very sick

6 = life-threateningly sick

Did such an infection lead to absence from work/school?

Yes

No

Unsure

If yes, the following additional question is asked:

How many days were you absent?

Dropdown from 1 to 90 days

**8l. Eye infection?**

No

Yes

If yes, the following additional questions are asked:

How many times in the last 3 months?

1 time

2 times

3 times

More than 3 times

Have you contacted a doctor for such an infection in the last 3 months?

Yes

No

Unsure

Have you received antibiotics for such an infection in the last 3 months?

Yes

No

Unsure

How many days have you felt sick from such an infection in the last 3 months?

Dropdown from 0 to 90 days

How sick did you feel at the worst?

1 = not sick

2 = slightly sick

3 = somewhat sick

4 = moderately sick

5 = very sick

6 = life-threateningly sick

Did such an infection lead to absence from work/school?

Yes

No

Unsure

If yes, the following additional question is asked:

How many days were you absent?

Dropdown from 1 to 90 days

**9. Do you have any of the following diseases?**

Diabetes: 1. No 2. Yes

High blood pressure: 1. No 2. Yes

Cardiovascular diseases: 1. No 2. Yes

Neurological diseases: 1. No 2. Yes

Pulmonary diseases: 1. No 2. Yes

Rheumatic diseases: 1. No 2. Yes

Cancer: 1. No 2. Yes

Allergies: 1. No 2. Yes

Sleep apnea: 1. No 2. Yes

**Thank you very much for filling out the questionnaire.**
