# Supplementary material for: Self-reported infections in Norway: a study of incidence, doctor visits, antibiotic use, and absence from work
Source: BMC Prim Care. 2026 Jul 17;27:364. doi: 10.1186/s12875-026-03478-0 (PMC13660301; doi:10.1186/s12875-026-03478-0)
Supplement: Supplementary file 2 [file 12875_2026_3478_MOESM2_ESM.docx]

**Additional file 2**

**Logistic regression analyses of experienced respiratory tract infection (no=0, yes=1) as the dependent variable (n=873).**

| Variables |  | Crude OR | Adjusted OR* | P-value |
| --- | --- | --- | --- | --- |
|  | | **(95% CI Lower-Upper)** | **(95% CI Lower-Upper)** |  |
| Sex | Male | 1.00 (Ref.) | |  |
|  | Female | **1.33 (1.02-1.74)** | 1.29 (0.95-1.74) | 0,100 |
| Age | 25-40 | 1.00 (Ref.) |  |  |
|  | 41-55 | **0.54 (0.38-0.76)** | **0.52 (0.35-0.77)** | **<0,001** |
|  | 56-70 | **0.50 (0.25-0.72)** | **0.57 (0.35-0.90)** | **0,016** |
| Marital status | Single | 1.00 (Ref.) |  |  |
|  | Married/cohabiting | 0.87 (0.61-1.26) | 0.84 (0.56-1.28) | 0,423 |
|  | Divorced/separated | 1.58 (0.84-2.98) | 1.67 (0.84-3.34) | 0,145 |
|  | Widow/widower | 0.91 (0.26-3.16) | 1.02 (0.26-3.94) | 0,978 |
| Country of birth | Norway | 1.00 (Ref.) | |  |
|  | Other European countries | 0.94 (0.57-1.55) | 0.81 (0.47-1.39) | 0,437 |
|  | Countries outside Europe | 1.11 (0.45-2.74) | 0.79 (0.30-2.10) | 0,635 |
| Children living at home | No | 1.00 (Ref.) | |  |
|  | Yes | **1.46 (1.11-1.92)** | 1.41 (0.69-2.89) | 0,343 |
|  | Children 0-2 year | **2.29 (1.24-4.24)** | 1.55 (0.76-3.16) | 0,262 |
|  | Children 3-6 year | **1.81 (1.09-2.99)** | 1.28 (0.71-2.30) | 0,425 |
|  | Children 7-12 year | 1.12 (0.78-1.59) | 0.78 (0.48-1.28) | 0,383 |
| Educational level | Primary school | 1.00 | |  |
|  | Secondary school | 1.39 (0.76-2.56) | 1.50 (0.77-2.94) | 0,232 |
|  | College/university | 1.66 (0.93-2.96) | 1.89 (0.99-3.59) | 0,053 |
| Chronic diseases |  | | |  |
| Diabetes | No | 1.00 (Ref.) | |  |
|  | Yes | 0.89 (0.45-1.67) | 1.06 (0.54-2.10) | 0,862 |
| Hypertension | No | 1.00 (Ref.) | |  |
|  | Yes | 0.87 (0.62-1.26) | 1.03 (0.69-1.54) | 0,895 |
| Cardiovascular diseases | No | 1.00 (Ref.) | |  |
|  | Yes | 0.92 (0.46-1.82) | 1.05 (0.51-2.18) | 0,897 |
| Neurological diseases | No | 1.00 (Ref.) | |  |
|  | Yes | 0.92 (0.54-1.57) | 0.89 (0.51-1.56) | 0,691 |
| Lung diseases | No | 1.00 (Ref.) | |  |
|  | Yes | **1.80 (1.02-3.18)** | **1.98 (1.09-3.59)** | **0,026** |
| Rheumatological diseases | No | 1.00 (Ref.) | |  |
|  | Yes | 1.22 (0.82-1.82) | 1.41 (0.92-2.16) | 0,114 |
| Cancer | No | 1.00 (Ref.) | |  |
|  | Yes | 0.67 (0.31-1.42) | 0.70 (0.32-1.57) | 0,390 |
| Allergies | No | 1.00 (Ref.) | |  |
|  | Yes | 1.21 (0.91-1.63) | 1.08 (0.79-1.48) | 0,616 |
| Sleep apnea | No | 1.00 (Ref.) | |  |
|  | Yes | 0.98 (0.62-1.54) | 1.14 (0.70-1.88) | 0,592 |
